# Construction of a feature gene and machine prediction model for inflammatory bowel disease based on multichip joint analysis

**DOI:** 10.1186/s12967-025-06838-z

**Published:** 2025-08-19

**Authors:** Yan Chaosheng, Sun Haowen, Rao Jingjing, Dai Yuanyuan, Duan Wenhui, Sheng Yingyue, Xue Yuzheng

**Affiliations:** 1 Department of Gastroenterology, Affiliated Hospital of Jiangnan University, 214122 Wuxi city, Jiangsu Province China; 2 Wuxi Medical College, Jiangnan University, 214062 Wuxi City, Jiangsu Province China

**Keywords:** Inflammatory bowel disease, Machine learning, Artificial neural network, Diagnostic model, Immune differences

## Abstract

**Background:**

Inflammatory bowel disease (IBD) is a chronic nonspecific inflammatory disorder triggered by immune responses and genetic factors. Currently, there is no cure for IBD, and its etiology remains unclear. As a result, early detection and diagnosis of IBD pose significant challenges. Therefore, investigating biomarkers in peripheral blood is highly important, as they can assist doctors in the early identification and management of IBD.

**Methods:**

We used a multichip joint analysis approach to explore the database thoroughly. On the basis of methods such as artificial neural networks (ANNs), machine learning techniques, and the SHAP model, we developed a diagnostic model for IBD. To select genetic features, we utilized three machine learning algorithms, namely, least absolute shrinkage and selection operator (LASSO), support vector machine (SVM), and random forest (RF), to identify differentially expressed genes. Additionally, we conducted an in-depth analysis of the enriched molecular pathways of these differentially expressed genes through Gene Ontology (GO) and Kyoto Encyclopedia of Genes and Genomes (KEGG) pathway enrichment analyses. Moreover, we used the SHAP model to interpret the results of the machine learning process. Finally, we examined the relationships between the differentially expressed genes and immune cells.

**Results:**

Through machine learning, we identified four crucial biomarkers for IBD, namely, LOC389023, DUOX2, LCN2, and DEFA6. The SHAP model was used to elucidate the contribution of the differentially expressed genes to the diagnostic model. These genes were associated primarily with immune system modulation and microbial alterations. GO and KEGG pathway enrichment analyses indicated that the differentially expressed genes demonstrated associations with molecular pathways such as the antimicrobial and IL-17 signaling pathways. By performing correlation and differential analyses between differentially expressed genes and immune cells, we found that M1 macrophages exhibited stable differential changes in all four differentially expressed genes. M2 macrophages, resting mast cells, neutrophils, and activated memory CD4 T cells all showed significant differences in three of the differentially expressed genes.

**Conclusion:**

We identified differentially expressed genes (LOC389023, DUOX2, LCN2, and DEFA6) with significant immune-related effects in IBD. Our findings suggest that machine learning algorithms outperform ANNs in the diagnosis of IBD. This research provides a theoretical foundation for the clinical diagnosis, targeted therapy, and prognostic evaluation of IBD.

**Supplementary Information:**

The online version contains supplementary material available at 10.1186/s12967-025-06838-z.

## Introduction

Inflammatory bowel disease (IBD) can be divided into ulcerative colitis (UC) and Crohn’s disease (CD). Since the emergence of IBD in the 20th century, the incidence rate of IBD has increased with industrialization and urbanization, similar to that of other immune-related diseases. Gabriel reported in the literature that the estimated prevalence of IBD affects approximately 5 million people and that there are approximately 400,000 new cases annually. In addition, age and sex are the key factors influencing the incidence rate and prevalence of IBD [1, 2]. For example, 25% of cases occur in childhood, and the incidence rate is increasing. Research indicates that IBD typically affects young people with relatively low mortality rates, and there is currently no curative treatment available [3, 4].

The increasing prevalence of IBD emphasizes the need for a deeper understanding of its molecular mechanisms to develop targeted therapies and diagnostic tools. Currently, in clinical practice, there are no biomarkers capable of accurately predicting the disease course or treatment response. This is attributed mainly to the complex molecular basis of IBD and variations in immune responses [5].

In reality, C-reactive protein (CRP), the erythrocyte sedimentation rate (ESR), fecal biomarkers, and calprotectin are often regarded as essential diagnostic tools for IBD. However, in practical applications, these so-called biomarkers have certain limitations. For example, fecal biomarkers have poor accuracy, the ESR is easily affected by multiple factors, and CRP production shows increased heterogeneity.

In recent years, with the data collected from genomic research, the relationships between specific genes and the etiology of IBD have been widely explored. As mentioned earlier, the association between the NOD2 gene and CD has been well established. It has become a key predictive factor and is associated with an increased risk of disease complications [6]. Additionally, individuals with genetic variability in the PRDM1 and NDP52 genes are more susceptible to CD. Genes such as KIF9-AS1, LINC01272, and DIO3OS have proven useful for differentiating and detecting various types of IBD. Regrettably, no research has yet been able to clearly explain the activity pathways of pathogenic genes and the immune cells affected by them.

Previous studies have indicated that IBD results from interactions among immune responses, genetic factors, and the microbiota [7]. Although the exact causes of IBD remain unclear, multiple interrelated factors, such as genetics, the immune system, the microbiota, and the environment, all play a role in the development of IBD [3]. Immune dysfunction can trigger persistent inflammation, a characteristic feature of IBD. This leads to a reduction in or destruction of intestinal crypts, along with a series of severe clinical manifestations and complications, significantly deteriorating the quality of life of patients [8, 9].

Our study revealed that, compared with normal controls, patients with IBD exhibited differential expression of several genes. These genes were enriched predominantly in pathways associated with inflammatory and immune responses, which is consistent with previous findings [10]. In addition to the differences in gene expression, we also discovered that the diagnosis of IBD could be reflected by changes in immune cells. We propose that differential gene expression serves as the initiating factor of IBD. Through several potential mechanisms, it acts on immune cells, leading to significant variations in their expression levels and quantities. First, differential gene expression may trigger excessive activation or inhibition of pathogenic molecular pathways in IBD patients, causing the release of an excessive amount of inflammatory factors. Second, the overexpression of inflammatory factors disrupts the immune response in patients. Eventually, the disordered immune response results in differential expression in immune cells in the body. In conclusion, differential gene expression plays a pivotal role in the initiation and progression of IBD and can be regarded as a key element in the development of novel diagnostic and therapeutic strategies for IBD. To explore this further, we used multichip joint analysis, artificial neural networks (ANNs), and machine learning algorithms to identify significantly differentially expressed genes. We then utilized the SHAP model to illustrate the contribution of these differentially expressed genes to the diagnosis. Finally, we performed correlation and differential analyses on immune cells to identify differences in gene expression among various immune cells.

## Materials and methods

### Data source, preprocessing, and analysis

In this study, we conducted a comprehensive analysis of multiple databases, including GEO, IBDDMB, and UKB. Specifically, we retrieved disease datasets (GSE87466 “www.ncbi.nlm.nih.gov/geo/query/acc.cgi? acc=GSE87466”, GSE179285 “www.ncbi.nlm.nih.gov/geo/query/acc.cgi? acc=GSE179285”, and GSE87473 “www.ncbi.nlm.nih.gov/geo/query/acc.cgi? acc=GSE87473”) from the GEO database. These datasets included data from 438 patients with inflammatory bowel disease and 51 healthy individuals. The patient biopsy data were sourced from the sigmoid colon, ascending/descending colon, and terminal ileum. The patient data included cases of moderate to severe active ulcerative colitis [11]. We utilized R software (version 4.3.3) for data preparation. During the preprocessing stage, we removed probes corresponding to multiple genes and converted probe IDs into gene symbols with the annotation file of the platform. When dealing with multiple probes for the same gene, we retained only the probe with the highest signal value. To ensure data consistency and reliability, we took measures to reduce the potential impact of batch effects, which are commonly introduced during the data integration process. Batch effects can occur due to variations in experimental conditions, instruments, or sample processing over time or across different datasets and may severely confound the interpretation of results. Thus, we used the “limma”, “pheatmap”, and “ggplot2” packages to calibrate the data for different groups (diseased and nondiseased). Additionally, the “pheatmap” and “ggplot2” packages were used to generate heatmaps and volcano plots of the differentially expressed genes (DEGs), respectively. The data were analyzed by log2 transformation.

### Transcriptome data refinement and analysis process

We utilized supplementary probe annotation files to convert the expression matrix from the probe level to the gene level. For genes associated with multiple probes, the arithmetic mean of the corresponding probe values was used to represent gene expression. Following this conversion, we standardized the dataset and then applied the SVA package for batch-effect correction. Principal component analysis (PCA) was utilized to assess the success of the standardization process. To identify the DEGs between the IBD and control samples, we used the limma package (linear model of microarray data). DEGs were defined as those with an absolute logarithmic fold change (|log FC|) greater than 2 and an adjusted *p* value less than 0.05. Particular emphasis was placed on genes that might be related to immune infiltration in IBD patients and normal individuals.

### Enrichment analysis

To clarify the biological significance and pathway associations of DEGs, we carried out comprehensive Gene Ontology (GO) and Kyoto Encyclopedia of Genes and Genomes (KEGG) analyses. In the R programming environment, we systematically explored the effects of differentially expressed protein-modifying genes (PMGs) on key biological processes (BPs), molecular functions (MFs), and cellular components (CCs). We used R software for this analysis, utilizing tools such as the “clusterProfiler” and “org” packages, along with the “Hs.eg.db”, “enrichplot”, and “ggplot2” packages. We enriched our interpretation with a focus on KEGG pathway data. This integrated approach allows us to observe the complexity of the molecular landscape of the IBD and the control groups, offering a comprehensive framework for further exploration of the underlying mechanisms.

### Artificial neural network (ANN)

ANN models play an increasingly crucial role in the realm of predictive modeling. This is because they are capable of capturing nonlinear relationships within high-dimensional datasets [12]. ANN models can predict complex variable relationships that other models, such as logistic regression models, are unable to achieve. The working principle of an ANN model is inspired by biological neural networks. In an ANN, each neuron is interconnected with other neurons. Neurons have two main components: dendrites and axons. Dendrites function as receivers of information, whereas axons serve as transmitters. The nucleus of a neuron holds the information to be transmitted. An ANN typically consists of an input layer, one or more hidden layers, and an output layer. Information enters the model through the input layer, undergoes processing in the hidden layer(s), and is then output via the output layer [13]. In our study, we utilized R software for correlation analysis. To increase the intuitiveness of the data, we used packages such as “neuralnet” and “NeuralNetTools” to generate relevant graphics.

### Machine learning algorithms

To identify candidate genes, we used the “VennDiagram” package to visualize the intersection of key genes among the DEGs. Three machine learning algorithms, namely, the least absolute shrinkage and selection operator (LASSO), support vector machine (SVM), and random forest (RF), were utilized to identify potential biomarkers. For the LASSO analysis, we used the “glmnet” package with a penalty parameter and 10-fold cross-validation. This approach was applied to select important variables from high-dimensional data. RF is an ensemble estimator that consists of multiple decision trees as its basic estimators. In the RF classification process, each tree determines a category, and the category receiving the highest number of votes is designated as the final output. The SVM considers each predictor as a dimension in a high-dimensional space. The SVM aims to find the optimal hyperplane for classifying samples and demonstrates excellent performance when dealing with highly complex data. Finally, the genes at the intersection of the results from the LASSO, RF, and SVM algorithms were identified as potential biomarkers for IBD.

### Explanation of machine learning models

Ten machine learning models were constructed with the selected predictive factors. These models included ridge least squares (RLS), RF, decision tree (DTS), SVM, logistic regression, k-nearest neighbors (KNN), extreme gradient boosting (XGBoost), gradient boosting machine (GBM), neural network, and generalized linear model boosting (GlmBoost). Some of these models possess high interpretability and are well suited for analyzing linear relationships. Other models are capable of capturing nonlinear relationships and interactions, making them suitable for handling high-dimensional data. For example, XGBoost is an optimized version of the gradient boosting framework. It supports parallel computing and can automatically handle missing values, showing excellent performance in clinical prediction tasks. We evaluated these models on the basis of several metrics, such as the receiver operating characteristic (ROC) curve, specificity, sensitivity, and accuracy, with the ROC curve serving as the primary evaluation indicator. The model that exhibited the best predictive performance was chosen as the main model for this study. To further understand the interpretability of the final prediction model, we used the Shapley sum interpretation (SHAP) method [14]. This method helps explain how each feature contributes to the model’s prediction, providing valuable insights into the underlying mechanisms of the model.

### Gene set enrichment analysis and gene set variation analysis

Gene set enrichment analysis (GSEA) is a powerful and widely used approach in genomics research. It aims to uncover biological pathways, functions, or molecular features associated with distinct phenotypes or experimental conditions. In this study, we downloaded the immunological signature gene set (c7: immunological signature gene set) and conducted GSEA on all genes within the immune cell cluster (version 1.64.0). Conversely, gene set variation analysis (GSVA) offers a unique way to represent gene set variation within a sample. It achieves this by converting gene expression data into gene set activity scores without the need for data ranking. To perform GSVA, we calculated the average expression value of genes in each cell cluster in the immune-related gene set (h: hallmark gene set) (version 1.50.0). Finally, we visually presented the results of the GSVA analysis. This visualization helps to better understand the differences and trends in gene set activities, providing valuable insights into the underlying biological mechanisms related to the immune cell clusters.

Immune Cell Infiltration and Correlation Analysis:

The CIBERSORT algorithm was used to assess the relative abundance of immune cells in each normal and IBD sample. Spearman correlation analysis was subsequently performed to determine the relationships between potential biomarkers and immune cells. Additionally, a differential analysis of immune cells was conducted for each gene individually.

### Data analysis

Each experiment was performed at least three times. The ROC curve was established, and the area under the curve (AUC) and 95% confidence interval (CI) values were calculated and validated with SPSS software. The statistical significance between the two groups was determined via Student’s t test. The results were further analyzed by GraphPad Prism version 7 software. A *P* value less than 0.05 was considered to indicate statistical significance.

Experimental verification: Real-time fluorescence quantitative PCR and Western blotting:

According to local legislation and institutional requirements, research on human participants requires ethical review and approval. In compliance with national legislation and institutional requirements, an ethics approval number for this study (LS2024012) was obtained. The IBD samples in this study were obtained from the remaining specimens of IBD patients who underwent pathological tissue diagnosis during their initial and follow-up visits. Enteritis samples were obtained from patients diagnosed with common enteritis. Normal human samples were sourced from healthy individuals undergoing physical examinations.

Western blotting (WB) was used to assess the protein expression levels of DUOX2, DEFA6, and LCN2 in 7 normal individuals, 4 patients with enteritis, and 9 patients with IBD, with GAPDH as the internal reference protein. Notably, LOC389023, which has been proven to be a pseudogene, is a long noncoding RNA located within chromosome 2q14.1 in the DPP10 gene; therefore, we did not perform Western blot detection on it. DUOX2 (Origene: 1000), LCN2 (Proteintech: 1000), DEFA6 (Proteintech: 1000), and GAPDH (Weiao: 1000) were used to quantify protein density with a Gel Pro analyzer.

The sample source for the RT‒PCR experiment was the same batch as for the second Western blotting sample, and the mRNA expression levels of DUOX2, DEFA6, LCN2, and LOC389023 were assessed in 4 normal individuals and 4 IBD patients. The primers used for amplifying DUOX2 were as follows: the F1 timer was gaagttcgacctcaggacca, and the R1 timer was agggagtgtgaagaagggctc. The primers used for DEFA6 were as follows: the F1 timer was aaccctcaccatccactg, and the R1 timer was cggcaaagtcctggtcattt. The primers for LCN2 were as follows: the F1 timer was gagggaagtggtatgtggt, and the R1 timer was tgcgggtctttgtcctct. The primers used for LOC389023 were as follows: the F1 timer was agcttcaatcagactgccct, and the R1 timer was gttccttccccatgtaccca. We selected GAPDH as the internal reference gene, with its forward primer being gcgagatccctccaaatcaa and its reverse primer being gttcacacccatgacgaacat. The sequence refers to the sequences of the target genes in the GenBank database, and the primers used were designed by NCBI Primer BLAST. GAPDH was used as the internal control. The reliability of the PCR results was verified to be consistent with the dissolution curve. The cycle threshold (Ct, which is the inflection point on the amplification power curve) was calculated, and the 2-ΔΔCt method [ΔCt = Ct (target gene) - Ct (GAPDH), ΔΔCt = ΔCt (target gene) - ΔCt (GAPDH)] was used to calculate relative gene expression.

## Results

### PCA data correction

**Fig. 1:**
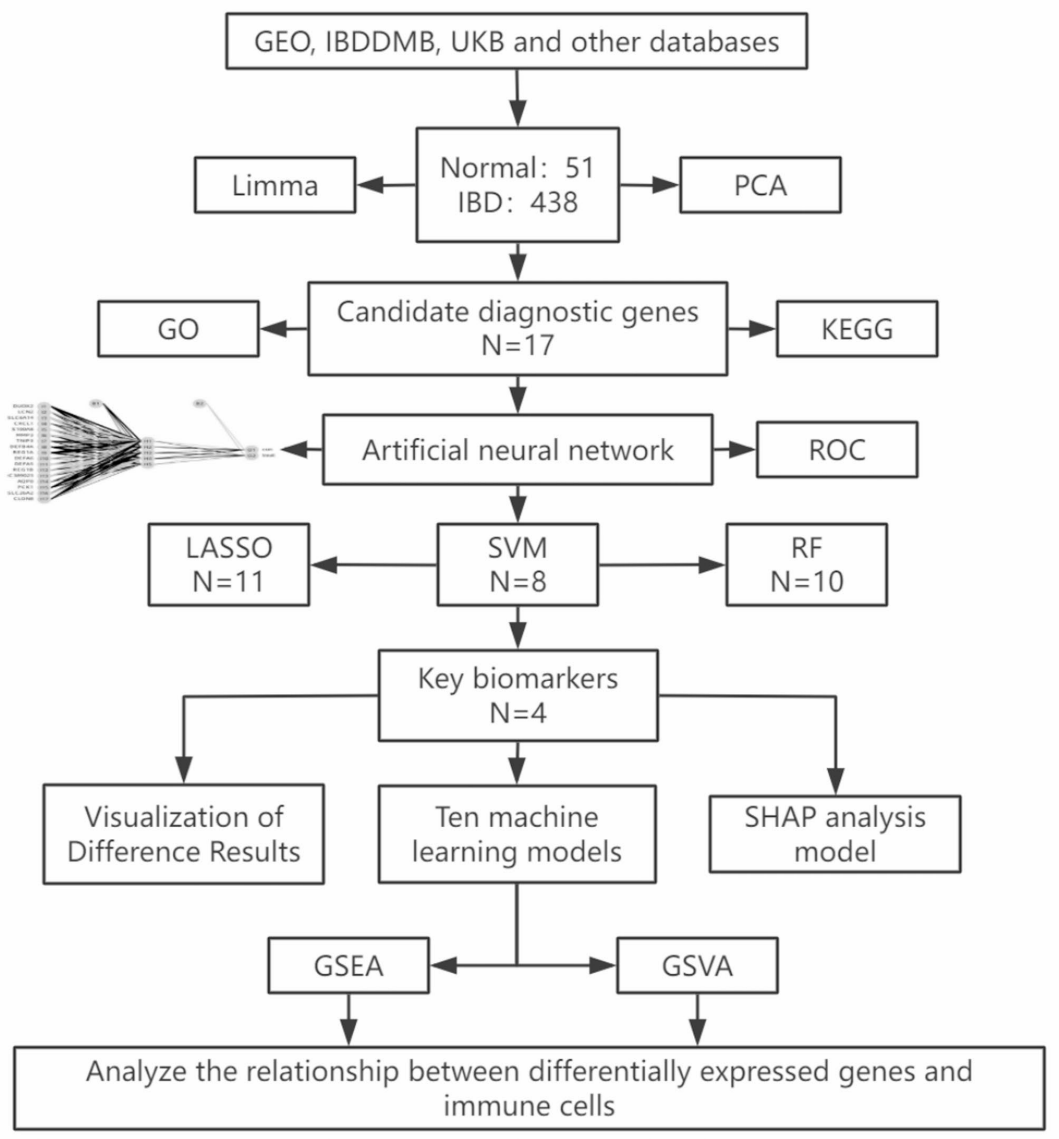
Flowchart of this study

**Fig. 2:**
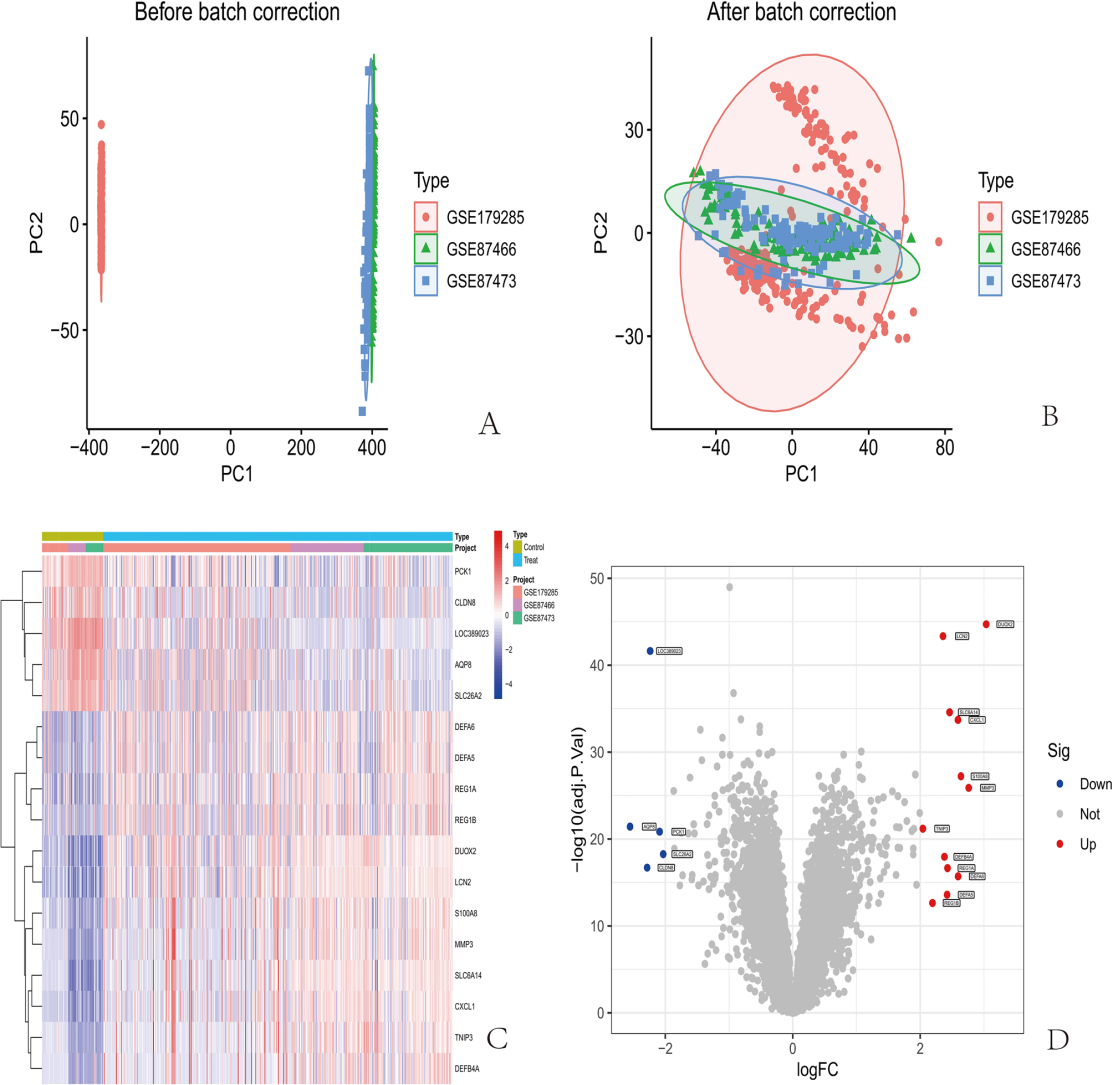
Analysis of the differences in gene expression between different datasets and the effectiveness of batch effect correction. (**A**) Distribution of samples from different experiments before batch calibration. (**B**) PCA involved randomly shuffling samples from different experiments. (**C**) Heatmap of DEGs in the IBD dataset. Red and blue indicate upregulated and downregulated DEGs, respectively. (**D**) Volcanic map of DEGs in the IBD dataset with |log2FC|>2. Red indicates an increase, and blue indicates a decrease

Figure 1 shows the flowchart of this study. Before batch calibration, samples from different experiments were separated, and a batch effect was observed between them. Through PCA, samples from different experiments were randomly shuffled to eliminate batch effects (Fig. 2A and B).

### Limma DEGs

The Limma method was used to identify DEGs in the GSE87466, GSE179285, and GSE87473 datasets, with 12 upregulated genes and 5 downregulated genes identified (Fig. 2C,

### Enrichment analysis results

**Fig. 3:**
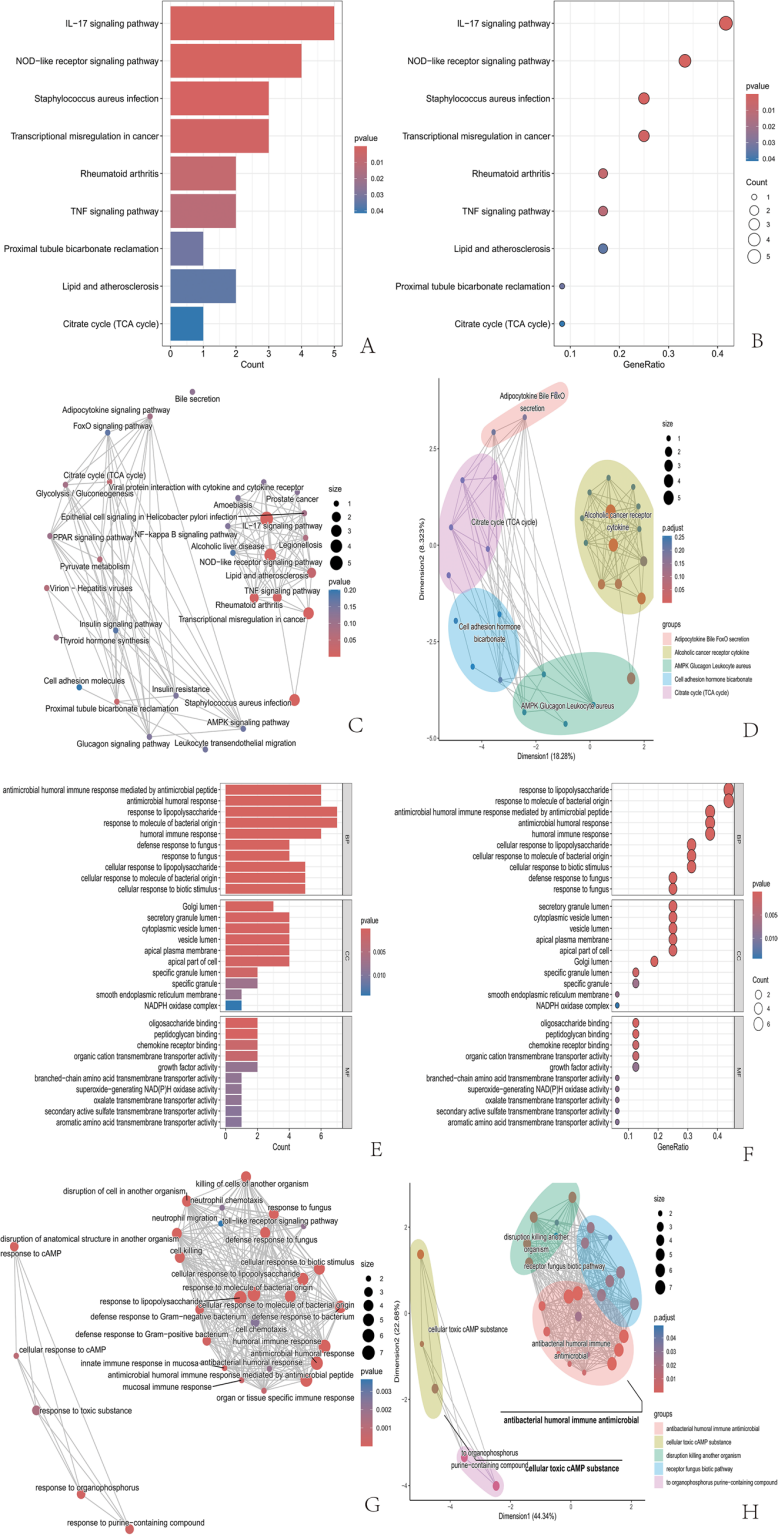
This set of images shows the analysis of DEGs, demonstrating different pathways and functional classifications. (**A-B**) KEGG pathway analysis, with the color becoming redder as the *P* value decreases. (**E-F**) GO analysis, including biological processes, cellular components, and molecular functions. The color becomes redder as the *P* value decreases. (**C, D, G, H**) Pathways enriched in the DEGs; the size of the dots indicates the number of DEGs contained in the corresponding pathways. The larger the number is, the larger the number of dots

The enrichment analysis of 17 candidate diagnostic genes revealed that they were predominantly associated with the immune response, microbial factors, and the inflammatory response, underscoring their crucial role in the pathogenesis and progression of IBD. KEGG analysis indicated that these genes were significantly enriched in categories such as adipocytokine secretion, bile acid-related pathways, the FOXO signaling pathway, the IL-17 signaling pathway, alcohol-related cancer-associated receptor-cytochrome interactions, and the AMPK-glucagon-leukocyte-*Staphylococcus aureus*-related pathways (Fig. 3A-D). In terms of biological processes, GO analysis revealed the involvement of these candidate genes in areas such as antimicrobial activity and cellular responses to toxic substances and cAMP (Fig. 3E-H). Moreover, molecular functional analysis revealed that the response to lipolysis was a significant category among these genes.

### ANN prediction results

**Fig. 4:**
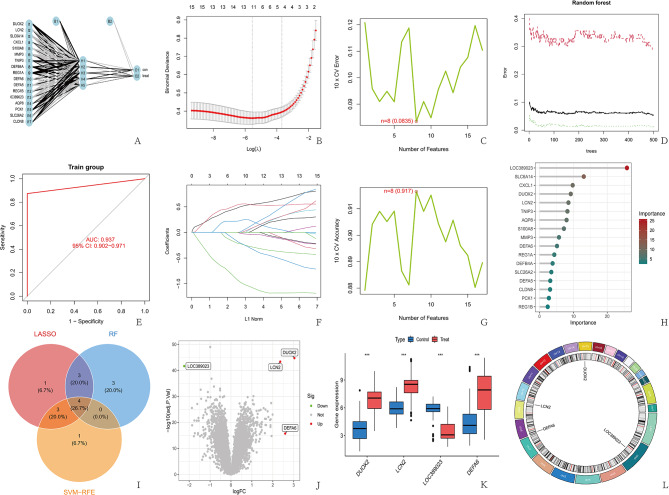
DEGs were screened through various methods, and the differential results were visualized. (**A, E**) The experimental group was distinguished from the control group with an ANN. A ROC curve was constructed to evaluate the overall diagnostic performance. (**B, F**) The genes were screened with the LASSO algorithm. To obtain the optimal model, a 10-fold cross-validation method was used. The lowest gene number (*n* = 11) at the lowest point of the curve was most suitable for LASSO. (**C, G**) Through the SVM algorithm for screening genes, accurate graphs and cross-validation error graphs were obtained, and 8 disease-characteristic genes were identified. (**D, H**) Screening genes through the random forest algorithm. Ten important genes were identified through the random forest method. IncNodePurity was used to sort genes on the basis of their relative importance. (**I**) The intersection of the results of the three algorithms yielded four genes. (**J**) Volcanic diagram of DEGs, with red indicating upregulation and green indicating downregulation. (**K**) Box plot of DEGs, with the horizontal axis representing the names of the intersecting characteristic genes and the vertical axis representing the expression levels of the genes. Blue indicates the sample of the control group, and red indicates the sample of the experimental group. (**L**) The outermost circle of the chromosome diagram represents the chromosome number, and the second circle represents the shape of the chromosome. The names of the intersecting genes are labeled at the corresponding positions on the chromosome

The ANN used in this study consisted of three layers: an input layer, a hidden layer, and an output layer. In the input layer, disease features and genes were assigned scores. Subsequently, on the basis of these scores and weights related to disease characteristics and genes, the hidden layer was generated. There were five nodes in the hidden layer, and the output layer was exported with these nodes and their respective weights. The output layer represents the properties of the sample. Result.matrix contained information such as training error (such as the mean square error (MSE)) and the number of iterations (epochs) that were used to evaluate training effectiveness. Weights stored the connection weights between all layers, which was used to analyze feature importance. Plotnet generated a network topology diagram that displayed the connection relationships among the input layer, hidden layer, and output layer. Prediction logic obtained the probability values of the model output through the compute() function and then converted them into category labels (con or treat) with which.max. The optimization algorithm was resilient back propagation (Rprop), which is an adaptive learning rate gradient descent algorithm. For loss function, by default, the least squares error (SSE) was used, which is suitable for regression problems but was used for classification (through probability output). The evaluation indicator used a confusion matrix to calculate the classification accuracy of the two groups. Unfortunately, the accuracy of our designed ANN in predicting the control group was only 21%, whereas the accuracy in predicting the experimental group reached 95.2%. To evaluate the overall diagnostic performance, we constructed ROC curves. The results revealed that the accuracy of the ANN for sample prediction was 93.7% (Fig. 4A and E).

### Selection results of the characteristic genes

We utilized LASSO regression to generate cross-validation graphs to identify the 11 disease-characteristic genes (Fig. 4B and F). Through the SVM method, we obtained accurate graphs and cross-validation error graphs, through which 8 disease-characteristic genes were identified (Fig. 4C and G). Through the RF method, a scoring map of forest trees and gene importance was obtained, and 10 disease-characteristic genes were selected (Fig. 4D and H). A Venn diagram was used to display the intersection of genes selected by the three machine learning algorithms. As a result, a total of four intersecting genes were identified (Fig. 4I).

### Visualization results

A box plot of the DEGs was constructed and combined with a volcano plot of the DEGs, which revealed that DUOX2, LCN2, and DEFA6 were upregulated in the experimental group and that LOC389023 was downregulated in the experimental group. The intersection of the characteristic genes and their distribution on the chromosome can be observed through the chromosome circle diagram (Figure 4J-L).

### Machine learning model results

**Fig. 5:**
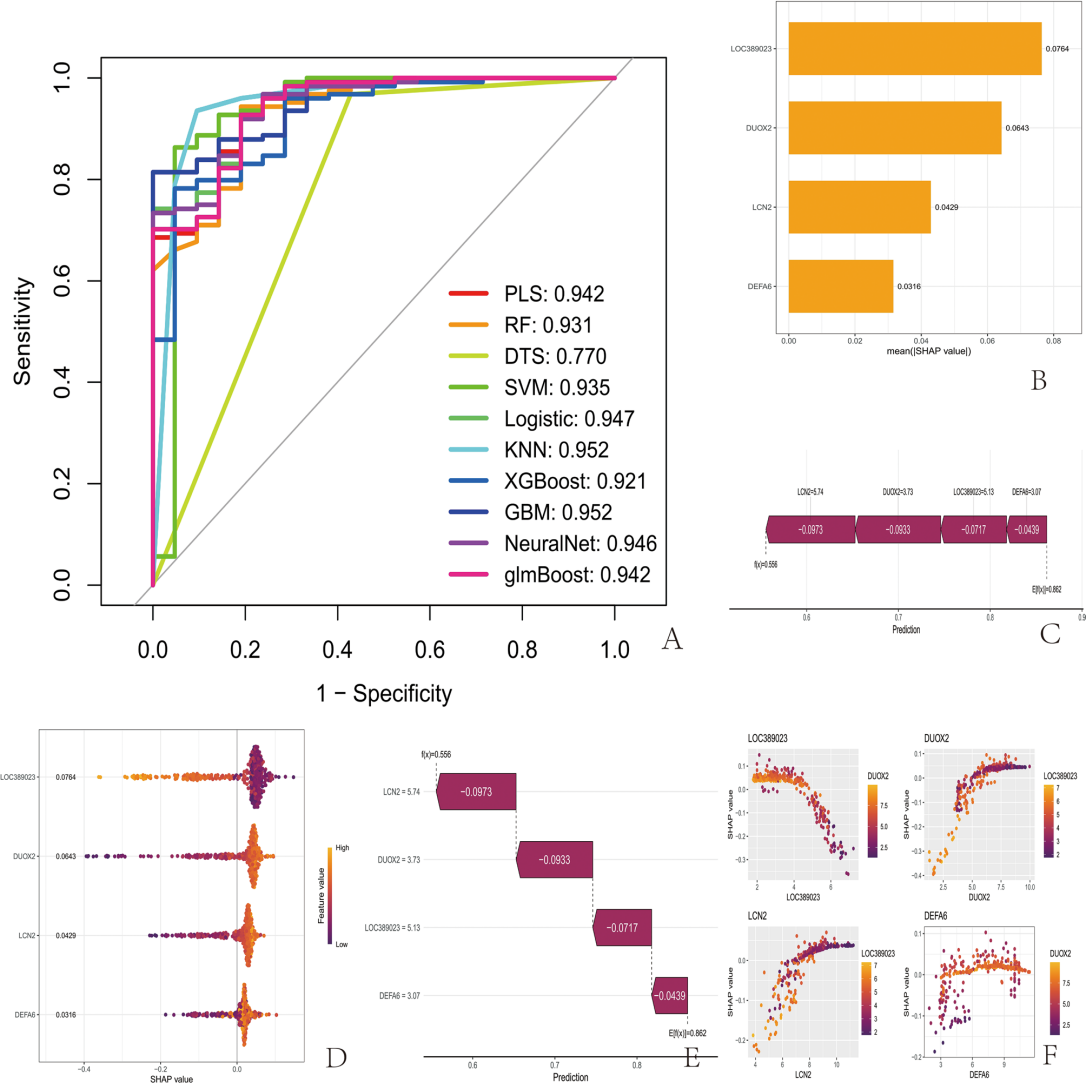
Prediction of the samples with machine learning models, and SHAP analysis of the machine learning models. (**A**) Ten machine learning models were used to construct ROC curves to evaluate the overall diagnostic performance. (**B**) Bar chart, with the vertical axis representing the gene name and the horizontal axis representing the mean absolute value of the SHAP value. The larger the value is, the more likely it is to indicate the gene and the greater the effect on the predicted results. (**C**) To present the results of the prediction of a single sample, the benchmark value was first determined, and then, for each gene, the result of the prediction was obtained. (**D**) The bee colony plot, with the vertical axis representing gene names and the horizontal axis representing SHAP values, allowed us to obtain the mean SHAP value for each gene. The larger the value is, the greater the contribution of that gene is. Each dot represents a sample, the color of the dot represents the gene expression level, with purple indicating low expression and orange indicating high expression. (**E**) Waterfall chart displaying the predicted results of a single sample. In this graph, the vertical axis represents the gene expression level, and the horizontal axis represents the predicted value. The larger the absolute value of the value is, the greater the effect of this gene on the predicted results. (**F**) Scatter plot in which the horizontal axis represents the expression level of one gene and the vertical axis represents the SHAP value. The dots represent the expression level of the gene, with purple indicating low expression and orange indicating high expression. The interaction relationship between these two genes and SHAP values can be observed

Through ten machine learning models, including RLS, RF, DTS, SVM, logistic, KNN, XGBoost, GBM, Neural Net, and GlmBoost, the diagnostic efficiency of the four IBD DEGs was evaluated through 10-fold cross-validation training control parameters. After the optimal diagnostic model was identified, the Type column of the training set was converted into binary labels, and the model was retrained with the optimal method. A ROC curve was constructed to evaluate the overall diagnostic performance (Fig. 5A).

### SHAP analysis results

By analyzing the predictive performance of machine learning, bar charts, bee colony plots, scatter plots, waterfall plots, and force plots were obtained. In the bar chart, the larger the value is, the greater the effect of this gene on the prediction results. The bee colony plot shows the mean SHAP value of each gene, which represents the contribution to the machine learning model. A scatter plot is a three-dimensional graph that allows observation of the interaction between genes and SHAP values. A waterfall plot can display the predicted results of a single sample, and the larger the absolute value of the value is, the greater the effect of this gene on the predicted results. To display the predicted results of a single sample, the benchmark value was first determined, and then, for each gene, the predicted result was obtained (Fig. 5B-F). In summary, through SHAP analysis, we explained the machine learning model and calculated the contribution size of each gene. In each sample, the patients could also be distinguished by gene expression. We interpreted the model by comprehensively analyzing the results of these genes.

### GSEA and GSVA results

**Fig. 6:**
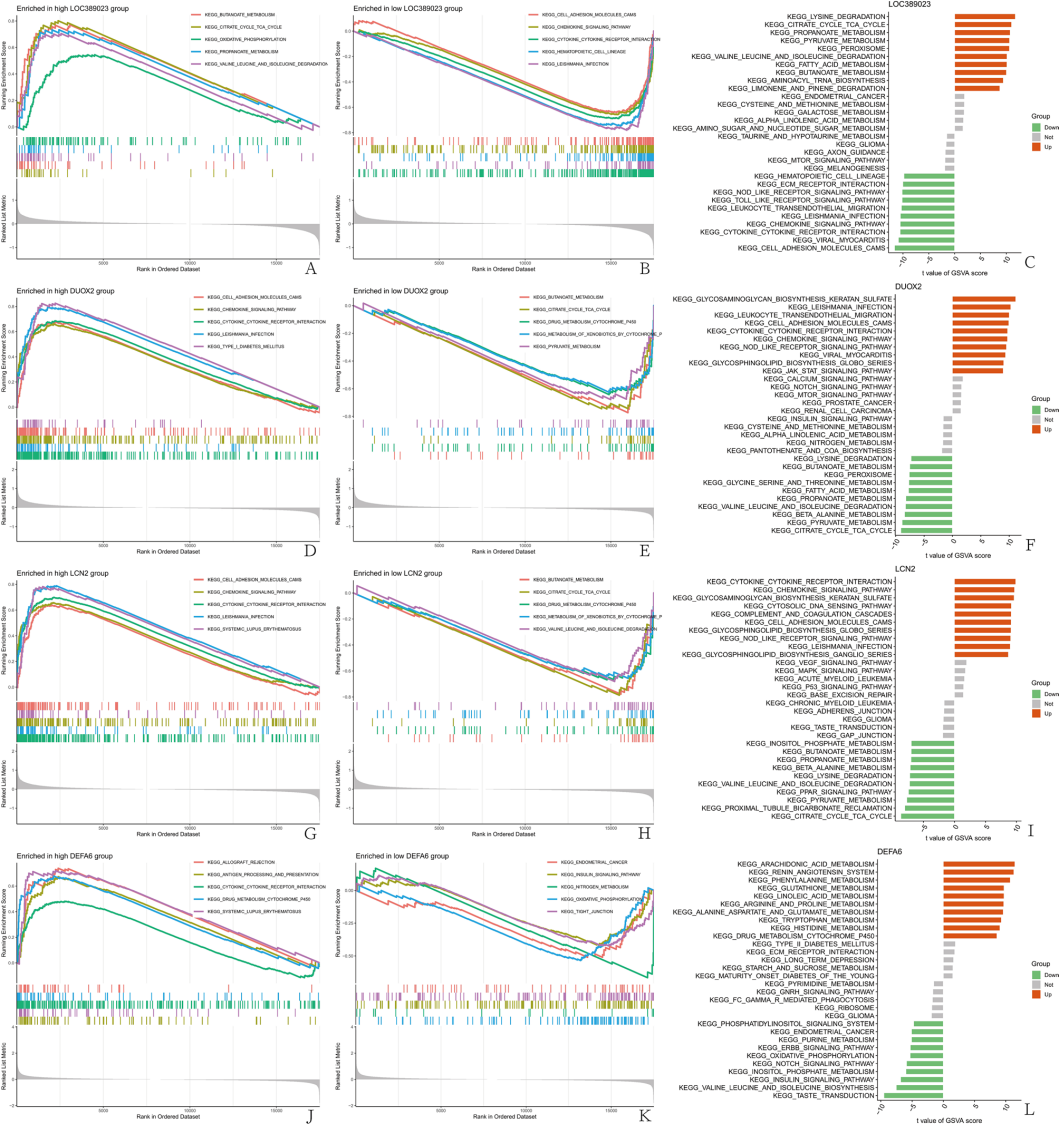
The GSEA/GSVA results of the target gene analysis were visualized. (**A, B, D, E, F, H, J, K**). The bar chart of the GSEA data, with the horizontal axis representing the sorted genes and the vertical axis representing the enriched scores, visualizes the top five pathways with the most significant enrichment. (**C, F, I, L**). In the GSVA bar chart, the vertical axis represents pathways, the horizontal axis represents T test values, red indicates upregulation of the target gene, and green indicates downregulation of the target gene. Gray indicates no difference in the target gene. (**A-C**) LOC389023, (**D-F**) DUOX2, (**G-I**) LCN2, (**J-L**) DEFA6

Through GSEA/GSVA, the functions or pathways enriched in the high-expression group or low-expression group of the target genes were analyzed, and the top five pathways with the most significant enrichment were visualized Figure 6A-L)

### Immune cell infiltration analysis results

**Fig. 7:**
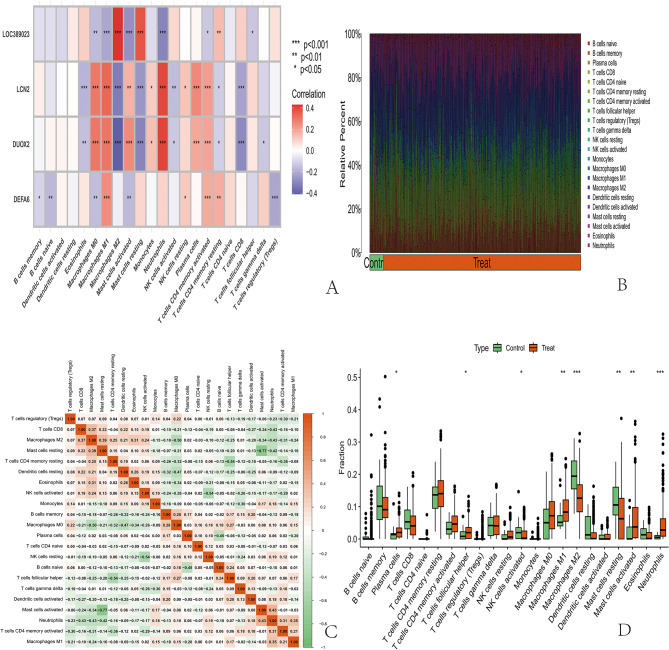
Immune-related analysis results. (**A**) Relationships between immune cells and target genes; *: *P* < 0.05, **: *P* < 0.01, ***: *P* < 0.001. (**B**) The number of immune cells in each sample was determined through immune cell infiltration analysis. The results of immune cell recordings were visualized with a bar chart, where the horizontal axis represents the sample and the vertical axis represents the content of the immune cells. The sum of all immune cells is one. Different colors indicate different immune cells. (**C**) The horizontal and vertical axes of the graph represent the names of the immune cells. The values inside represent the correlation coefficient, with red indicating a positive correlation and green indicating a negative correlation. (**D**) Box plot of the differences, with the horizontal axis representing the names of immune cells and the vertical axis representing the content of immune cells. Green indicates the samples of the control group, and red indicates the samples of the experimental group. *: *P* < 0.05, **: *P* < 0.01, ***: *P* < 0.001

**Fig. 8:**
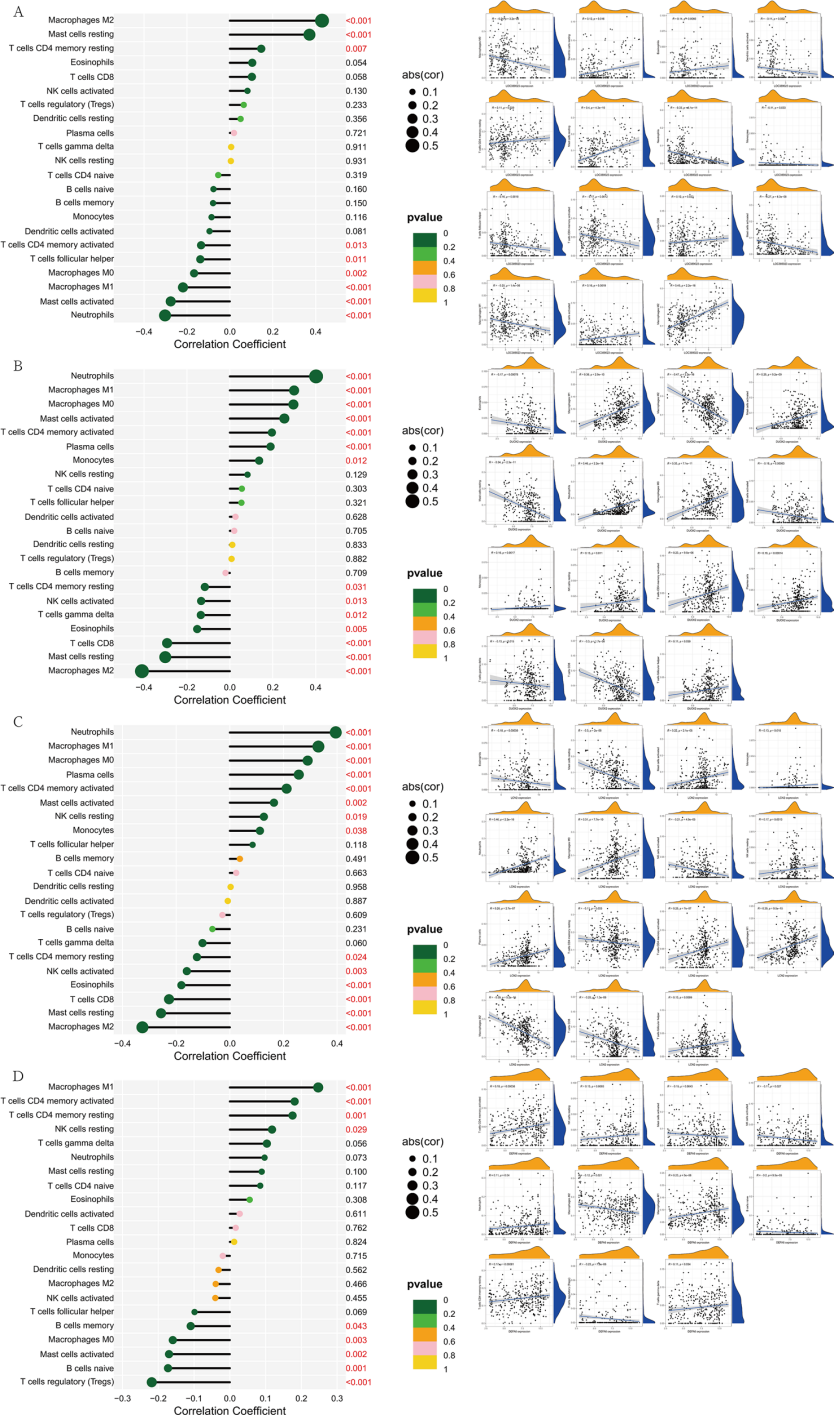
Correlation analysis between differentially expressed genes and immune cells. (**A**) LOC389023, (**B**) DUOX2, (**C**) LCN2, and (**D**) DEFA6. Correlation lollipop charts in which the vertical axis represents the names of immune cells, the horizontal axis represents the correlation coefficient, the size of the circle represents the absolute value of the correlation coefficient, and the color of the circle represents the *P* value of the correlation test. Scatter plots in which the horizontal axis represents the expression level of the target gene, the vertical axis represents the content of immune cells, the R value represents the correlation coefficient, and the *P* value represents statistical validity

The content of immune cells in each sample was determined through immune cell infiltration analysis, and the results were visualized to obtain a bar chart. Notably, the content refers to the relative content, and the sum of all immune cells is one. In the box plot of differences, an asterisk above a certain immune cell indicates that there were differences in that immune cell type between the control group and the experimental group. The horizontal and vertical axes of the correlation graph represent the names of the immune cells, and the values inside represent the correlation coefficients (Fig. 7A-D). To investigate the relationships between immune cells and LOC389023, DUOX2, LCN2, and DEFA6, scatter plots were generated for immune cells with significant correlations. Then, we visualized the correlation results and obtained correlation lollipop plots (Fig. 8A-D).

### Experimental results

We collected 3 samples of normal human intestinal tissue, 2 samples of intestinal tissue from patients with enteritis, and 5 samples from patients with IBD. The presence of target proteins in the sample was detected through WB, and quantitative analysis and measurement of grayscale values for the target proteins were performed.

DUOX2, with a molecular weight of approximately 130 kDa, LCN2, with a molecular weight of 25 kDa, and DEFA6, with a molecular weight of 11 kDa, were detected in all three groups. Visual inspection of the bands revealed differences in expression levels between the groups, but there was a similar trend between the colitis group and the IBD group. Therefore, we conducted one-way ANOVA to test whether multiple population means were equal, analyzed intergroup and intragroup variations, and determined whether there were significant differences in the relative expression levels of target proteins between different groups. Although no obvious trend was observed in the visual bands, one-way ANOVA of the ratio of the target protein grayscale value to the internal reference protein GAPDH grayscale value revealed significant differences in the target protein among normal individuals, enteritis patients, and IBD patients.

**Fig. 9:**
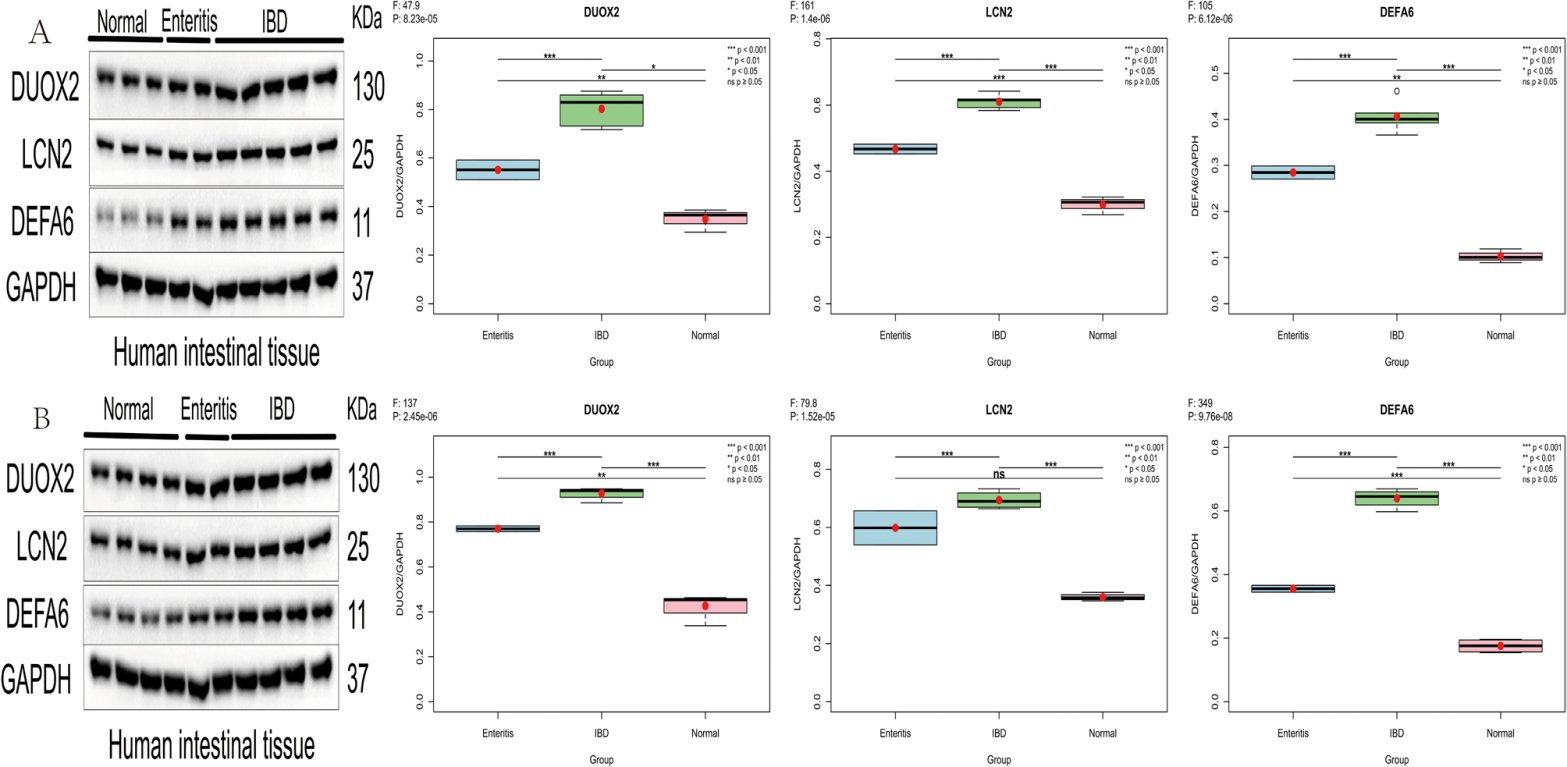
Detection of DUOX2, LCN2, and DEFA6 expression in 20 frozen human intestinal tissues with WB (**A**: first WB experiment, **B**: second WB experiment)

To eliminate the randomness of the experiment and reduce experimental errors, we reselected 4 samples of normal human intestinal tissue, 2 samples of intestinal tissue from patients with enteritis, and 4 samples of intestinal tissue from patients with IBD. We repeated the above experiment and found that the results were not significantly different from those of the first experiment. The experimental data revealed good discrimination for DUOX2, LCN2, and DEFA6 in normal individuals, enteritis patients, and IBD patients (Fig. 9).

**Fig. 10:**
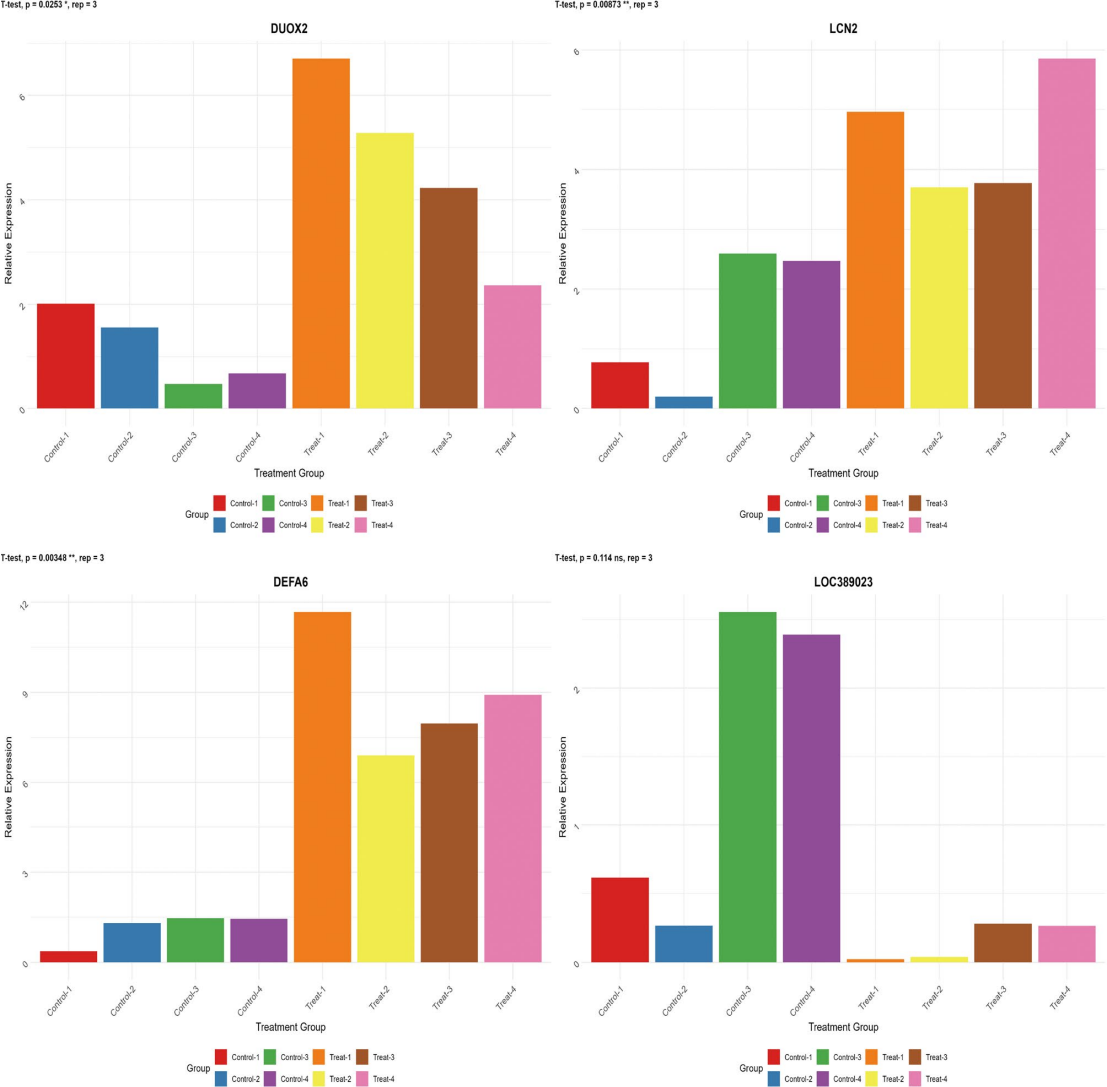
The 2^−ΔΔCt^ values of different genes (DUOX2, LCN2, DEFA6, and LOC389023) in multiple sample groups were determined by real-time PCR. The vertical axis represents relative gene expression levels, whereas the horizontal axis represents grouping information (*p < 0.05, **p < 0.01, ***p < 0.001)

We conducted real-time PCR experiments using samples from the second WB experiment, compared the expression levels of different genes in each sample group, and analyzed the expression characteristics and differences of each gene under different conditions. The experimental data revealed good discrimination for DUOX2, LCN2, and DEFA6 between normal individuals and IBD patients and poor discrimination for LOC389023 between normal individuals and IBD patients (*P* = 0.114) (Fig. 10).

## Discussion

Our study represents a comprehensive genetic-based interpretation of the differences in genes, activity pathways, and immune responses in IBD. We integrated multichip analysis, ANN, and ten machine learning methods. Additionally, we performed interpretable analysis on the machine learning model to explore the contribution of DEGs to the diagnosis of IBD. By using DEGs as a benchmark, we investigated the changes in each gene in the immune response and examined the alterations in and correlations among immune cells. This allowed us to elaborate on the genetic factors of IBD patients from both genetic and immune response perspectives. On the basis of these findings, we developed a predictive model and validated it multiple times with data from the GEO database.

IBD results from the interaction between immune responses and genetic factors, but the effects of factors such as microorganisms, the environment, and diet cannot be ignored [15]. Currently, the clinical diagnosis of IBD is still restricted by various limitations, and a simple and convenient diagnostic method remains to be developed. The biomarkers identified through our screening can be detected in peripheral blood, enabling easy assessment of the likelihood of IBD in subjects [16, 17, 18].

In our study, we successfully identified four biomarkers for the diagnosis of IBD, namely, LOC389023, DUOX2, LCN2, and DEFA6. These biomarkers play crucial roles in immune function. The ANN we designed exhibited an accuracy rate of merely 21% for predicting in the control group, yet it achieved a remarkable 95.2% accuracy rate for predicting in the experimental group. By constructing ROC curves to assess the overall diagnostic performance, the accuracy of sample prediction with the ANN reached 93.7%. We utilized these biomarkers to construct machine learning models. After selecting the optimal model through 10-fold cross-validation, we transformed the raw data for secondary validation. Among the machine learning algorithms, GBM and KNN demonstrated the highest diagnostic performance, reaching 95.2%. They were able to distinguish effectively between the experimental and control groups. However, there were numerous uncertainties regarding the diagnostic performance of the ANN. We made multiple attempts to modify the number of hidden layers and conducted small-scale validations on the data. Unfortunately, the results did not meet our expectations. After consulting the relevant literature and experts, our general hypothesis was that gene scoring might have led to data overfitting in the ANN. Nonetheless, we used various types of machine learning methods to analyze the data from multiple dimensions and perspectives, which effectively circumvented these issues. To this end, we compared the results of the ANN and machine learning models, which highlighted that the machine learning models were more reliable. Nevertheless, large-scale validation is still needed to confirm their generalizability.

We assessed the potential of LOC389023, DUOX2, LCN2, and DEFA6 as diagnostic biomarkers through real-time fluorescence quantitative PCR and Western blot experiments. In our study of diagnostic biomarkers for IBD, we initially included patients with common enteritis as normal individuals in the sample, on the basis of a clear distinction between the two diseases in clinical practice. Common enteritis has different etiologies and relatively good prognoses and is fundamentally different from IBD. However, the Western blot results revealed that patients with common enteritis and patients with IBD exhibited the same trend, which was indeed unexpected. We conducted secondary experiments and reselected samples, and the results revealed that DUOX2, LCN2, and DEFA6 had good diagnostic efficacy. We comprehensively analyzed the data and found that the genes we screened are related to immunity, and thus, the increased expression in the enteritis group is also of practical significance. Moreover, the genes we screened exhibited good discrimination in normal individuals, enteritis patients, and IBD patients, which is a new discovery in our experiments. On the basis of the WB results, we conducted real-time fluorescence quantitative PCR experiments on samples from normal individuals and IBD patients for the second time and found that DUOX2 (*P* = 0.0253), LCN2 (*P* = 0.00873), and DEFA6 (*P* = 0.00348) still demonstrated outstanding discrimination, whereas LOC389023 performed poorly (*P* = 0.114), which may be related to its nature as a long noncoding RNA. It cannot be ruled out that our experiment may have errors.

Research findings have indicated that LOC389023 plays a vital role in regulating gene expression and chromatin status. LOC389023 is a long noncoding RNA located within chromosome 2q14.1 and the DPP10 gene. LOC389023, in the nuclei of neurons, contains GC-rich stem-ring motifs. These motifs can bind to the SUZ12 protein in the chromatin and the polycomb repressive chromatin-modifying complex 2 (PRC2) complex. By doing so, it recruits chromatin-remodeling inhibitors, leading to a reduction in DPP10 gene expression. This regulatory effect exhibits cell type specificity. This study also revealed the relationships among LOC389023, gene expression, histone methylation, and voltage-gated K(+) channels during neural development [19]. DNA methylation and noncoding RNAs have been extensively investigated in patients with IBD. DNA methylation is dependent on dietary cofactors such as substrates and nutrients (folate, vitamin B12/D, etc.). It is associated with inflammation, microbiota composition, and microRNAs, which can influence IBD by interfering with T-cell differentiation. Marangoni et al. conducted a comprehensive analysis of the role of DNA methylation in IBD and its effect on the inflammatory process [20]. Furthermore, studies have revealed that voltage-gated K(+) channels, such as KV1.3 and KCa3.1, are involved in K(+) conductance in T lymphocytes. They play crucial roles in cell proliferation, differentiation, apoptosis, and infiltration. In chemically induced IBD model mice, the activity and expression of KCa3.1 in CD4(+) T lymphocytes of mesenteric lymph nodes were increased. Moreover, its regulatory factor NDPK-B was positively expressed. When the KCa3.1 K(+) channel was blocked with TRAM-34 and/or ICA17043, the severity of IBD was significantly reduced. Symptoms such as diarrhea, fecal blood, inflammation, and colonic crypt injury were alleviated, and simultaneously, the expression levels of KCa3.1 and Th1 cytokines in CD4(+) T lymphocytes were restored. These findings indicate that abnormalities in the KCa3.1 channel are related to the development of IBD and that interventions targeting this channel can improve disease conditions [21, 22]. In addition, the upregulation of K2P5.1 in T lymphocytes is associated with the pathogenesis of autoimmune diseases. Given that IBD also belongs to this category of diseases and that ion channel pre-mRNA splicing is related to disease, the mRNA splicing mechanism regulated by K2P5.1 K(+) channel transcription holds guiding significance for the treatment of IBD and other diseases [22].

Lipocalin 2 (LCN2), a member of the adipokine protein family, is predominantly involved in processes such as cell growth, differentiation, metabolism, and the immune response. During inflammation, various cell types, including macrophages, epithelial cells, and neutrophils, secrete LCN2. It then exerts its effects on other cells via the bloodstream or the local tissue microenvironment, thereby influencing inflammatory responses, immune regulation, and other physiological processes. LCN2 is implicated in both acute and chronic inflammation and plays crucial pathogenic roles in diseases such as cancer, diabetes, obesity, and multiple sclerosis [23, 24]. Through a series of cell- and tissue-based studies, Xia et al. discovered that LCN2 is key in regulating iron homeostasis and the inflammatory response. LCN2 can interact with iron and iron carriers in diverse cell types, including immune cells and epithelial cells. By binding to bacterial iron carriers, it inhibits bacterial growth. Moreover, it can regulate cell survival, apoptosis, and other processes by modulating intracellular iron levels. Under inflammatory conditions, LCN2 stabilizes the iron pool and reduces iron-related toxicity. For example, in intestinal inflammation, LCN2 restricts the availability of iron in the intestine, safeguarding the mucosa from damage. In kidney diseases, it protects against acute kidney injury, yet in chronic kidney disease, it may exacerbate disease progression. Additionally, LCN2 has a complex role in tumor cells. It can both promote tumor cell growth and metastasis and potentially inhibit tumor development [25–27]. Qun et al. conducted experiments in which mice were infected with harmful bacteria and reported that LCN2 is involved in microbial invasion, the inflammatory response, and tissue damage. It also participates in regulating the balance of the gut microbiota and its metabolites. When the LCN2 gene is absent, significant changes occur in the operational taxonomic units (OTUs) and alpha- and beta-diversity of the gut microbiota in mice infected with harmful bacteria. Simultaneously, intestinal metabolites are affected, with increased levels of metabolites such as taurodeoxycholic acid and undecylenic acid. These findings indicate that LCN2 may modulate the intestinal environment by regulating the composition and metabolite levels of the gut microbiota, thus influencing host health and disease resistance [28].

There are two types of dual oxidase (DUOX) enzymes, namely, DUOX1 and DUOX2. Their primary function is to generate reactive oxygen species (ROS) in tissues such as the thyroid, colon, respiratory tract, and lymphatic system. DUOX significantly contributes to the synthesis of hydrogen peroxide (H_2_O_2_), a substance that plays a pivotal role in the host defense system. H_2_O_2_ is involved in processes such as signal transduction, cell differentiation, cell death programs, immune defense, microbial composition regulation, and hormone synthesis (specifically that of thyroid hormones) [29, 30]. Helmut carried out a multiomics whole-phenotype association study (PheWAS) on 2872 participants to analyze the relationship between DUOX2 gene variations and the IBD phenotype. This study revealed that rare variations in the DUOX2 gene were associated with an elevated risk of IBD. Through multiomics PheWAS and rare variation association analysis, the link between DUOX2 variations and the pathogenesis of IBD was elucidated [30]. In the context of respiratory diseases, with respiratory viral infections as an example, Ducquin demonstrated that DUOX2 selectively regulates the cytokines and chemokines secreted by epithelial cells. This, in turn, affects the recruitment, adhesion, and degranulation of neutrophils, revealing that DUOX2 plays a crucial role in modulating the interaction between epithelial cells and immune cells [31, 32]. In studies of congenital hypothyroidism (CH), DUOX2 mutations have been extensively explored. In a critical CH cohort, 50% of patients carried DUOX2 mutations (38%). These mutations are associated with patients’ biochemical characteristics, which influence the diagnosis and treatment of the disease. Moreover, current screening thresholds may result in missed diagnoses. At the cellular level, the expression of DUOX2 on the cell membrane and its mediation of H_2_O_2_ production are highly important. When DUOX2-mediated H_2_O_2_ production is completely lost, it further impairs the synthesis of thyroid hormones, ultimately leading to the development of CH. From the perspective of signaling pathways, Juan’s research indicated that DUOX2 plays a significant role in the development of IBD-related tumors. When the TLR4 signaling pathway is activated in epithelial cells, the expression of DUOX2 is upregulated. DUOX2 then catalyzes the production of H_2_O_2_, which is closely associated with the initiation and progression of tumors. Additionally, DUOX2 interacts with the microbiota. In this process, the generated H_2_O_2_ promotes tumor development, affecting the transition from IBD to tumors [33, 34].

DEFA6 is secreted by Paneth cells, specialized epithelial cells located at the base of the small intestine crypts, and represents the most abundant antibacterial agent produced by Paneth cells in the small intestine. Its release into the crypt lumen is thought to safeguard against microbial invasion into the crypt microenvironment [35]. As an antimicrobial peptide, DEFA6 plays a crucial role in the intestinal immune defense system by combating pathogens. A study was conducted a study on mucosal samples obtained from 88 CD patients who underwent ileocolonic resection. The results revealed that while the expression of DEFA6 in the healthy and diseased ileal mucosa of early- and late-stage CD patients did not significantly differ, there was an increasing trend in the expression of DEFA6 in the external validation cohort of late-stage CD patients. As the course of CD progresses, the expression of antimicrobial peptide-related genes, such as DEFB4A, increases in the affected mucosa. It is hypothesized that persistent mucosal damage may enable intestinal bacteria to interact with epithelial cells, thereby stimulating the expression of antimicrobial peptides. Abundant evidence indicates that alterations in DEFA6 expression are associated with the disease course of CD [36]. Stephen’s research revealed that DEFA6, which serves as a specific marker for Paneth cells in the small intestine, contributes to intestinal immune defense and the maintenance of the gut microbiota balance [37, 38]. Serena’s work confirmed that DEFA6 functions to maintain intestinal immune homeostasis in the Paneth cells of human small intestine organoids. Studies have shown that Paneth cells can produce antibacterial substances such as DEFA6 to preserve the gut microbiota balance. However, the expression level of DEFA6 in human small intestine organoids is extremely low and significantly differs from that in the source tissue. In contrast, mouse small intestine organoids can more effectively mimic the expression of α-defense factors in tissues. Moreover, scientists have reported that WNT signal stimulation fails to restore the expression of DEFA6 in human small intestine organoids. Nevertheless, after treatment with FOXO inhibitors, the mRNA expression of DEFA6 increased by more than 100,000-fold, nearly reaching the level in human tissue. These findings indicate that the FOXO signaling pathway is essential for regulating the expression of DEFA6 in human Paneth cells. Inhibiting the FOXO signaling pathway can effectively restore the expression of DEFA6, which is highly important for the study of intestinal diseases and the enhancement of intestinal immunity [39].

GO and KEGG analyses revealed that the DEGs were predominantly enriched in the antibacterial aspects of the human immune system and the IL-17 signaling pathway. Nicholas conducted a study on 136 IBD patients and reported that patients with low IgG/G1 levels had poorer clinical survival data than did those with normal levels. These findings suggest that humoral immunity plays a pivotal role in the survival of IBD patients. When IBD patients experience compromised humoral immunity, their likelihood of requiring surgery increases. This implies that low IgG/G1 levels have differential effects on the surgical requirements of different subtypes of IBD patients. These findings also indicate that humoral immunity can serve as a predictor of IBD patient survival, which is highly important for their clinical management. IL-17 is closely associated with IBD and is essential in the pathological progression of IBD. Research has demonstrated that IL-17 is a key cytokine secreted by Th17 cells and plays a substantial role in the development of intestinal inflammation in IBD patients [40]. In CD patients, IL-17-producing cells accumulate in large quantities in the submucosal and muscularis propria layers. Moreover, compared with that in healthy individuals, the number of IL-17-producing T cells in CD patients is significantly greater. Ample evidence points to a strong link between IL-17 and IBD [41]. Furthermore, Kosaku’s research has also shown that IL-17 is closely related to IBD and plays a crucial role in its pathogenesis [42, 43]. Through whole-exome sequencing analysis of colon organoids from UC patients and healthy controls, it was discovered that the UC inflammatory epithelium accumulates somatic mutations in multiple genes associated with the IL-17 signaling pathway, such as NFKBIZ, ZC3H12A, and PIGR. These genes are rarely affected in colon cancer but are mutated within the inflammatory environment of UC. These findings indicate that the IL-17 signaling pathway is significantly perturbed in the pathological process of UC. Additionally, gene mutations related to the IL-17 signaling pathway may also be implicated in the occurrence and development of UC in humans. They may disrupt the intestinal immune balance by interfering with the IL-17 signaling pathway, thus promoting the progression of UC [43].

Our research revealed differences in plasma cells, follicular helper T cells, activated natural killer (NK) cells, M1 macrophages, resting mast cells, activated mast cells, M0 macrophages, and neutrophils between the control group and the experimental group. Through a subsequent investigation of the relationships between genes and immune cells, we revealed that there were significant differences in LOC389023 in M1 macrophages, M2 macrophages, activated mast cells, resting mast cells, and neutrophils. Significant differences in LCN2 were observed in eosinophils, M0 macrophages, M1 macrophages, M2 macrophages, resting mast cells, neutrophils, plasma cells, activated CD4 memory T cells, and CD8 T cells. Stronger associations of DUOX2 with M0 macrophages, M1 macrophages, M2 macrophages, activated mast cells, resting mast cells, neutrophils, plasma cells, activated memory CD4 T cells, and CD8 T cells were detected. For DEFA6, significant changes were noted in M1 macrophages, activated memory CD4 T cells, and regulatory T cells (Tregs). Moreover, M1 macrophages exhibited consistent differential changes when the four DEGs varied. M2 macrophages, resting mast cells, neutrophils, and activated memory CD4 T cells all presented obvious differences in three of the DEGs. Research by Chen revealed that macrophages can polarize into two phenotypes, M1 and M2. M1 macrophages are involved mainly in pro-inflammatory responses and can secrete pro-inflammatory factors such as IL-6, IL-12, and TNF. M2 macrophages are involved mainly in anti-inflammatory responses and contribute to tissue repair, with the characteristic expression of arginase-1 (Arg-1), mannose receptor (CD206), and the anti-inflammatory factor IL-10. IBD is an intestinal inflammatory disorder, and the inflammatory microenvironment in the intestine is closely related to macrophage polarization [44, 45]. On the basis of this author’s research findings, we speculate that the overactivation of M1 macrophages may exacerbate the intestinal inflammatory response in IBD patients, leading to tissue damage. In contrast, the anti-inflammatory and tissue-repair functions of M2 macrophages may help alleviate the inflammatory symptoms of IBD and promote the repair of intestinal tissue. Regrettably, none of these issues have been verified, and therefore, it cannot be simply stated that M1/M2 macrophages are closely associated with IBD. However, through bioinformatics analysis methods and the use of GEO data, the results of our comprehensive analysis indicate that macrophage polarization plays an important role in IBD [45].

Zhen’s research revealed that, in colorectal cancer patients, the density of mast cells is lower than that in normal tissues, and their phenotype undergoes substantial changes, shifting from a quiescent state to an activated state. In the tumor microenvironment, activated mast cells release a variety of bioactive substances, including histamine, cytokines, proteases, and lipid mediators, which are capable of triggering inflammatory responses [46]. On the basis of these findings, we hypothesize that the abnormal expression of DEGs leads to the overactivation of immune cells. These activated immune cells then release active substances, thereby contributing to the onset and progression of IBD. Our findings indicate that mast cells exhibit significant differences in the expression of numerous DEGs [47].

Neutrophils play a key role in the innate immune system of the intestine [48]. Camille’s study demonstrated that neutrophil infiltration serves as a marker of disease activity in IBD patients. Neutrophils can release various inflammatory mediators, such as ROS, cytotoxic particle contents, and neutrophil extracellular traps (NETs), thereby triggering inflammatory responses and causing tissue damage [49, 50]. However, simultaneously, neutrophils are also essential for maintaining the intestinal barrier, host defense, and reducing inflammation. For example, CD177 + neutrophils have a protective effect. This study also revealed that neutrophil infiltration is associated with disease severity and that excessive neutrophil infiltration may lead to treatment failure. In CD, the dysfunction and reduced recruitment of neutrophils result in delayed bacterial clearance and the persistent presence of antigens, which trigger adaptive immune responses and the formation of granulomas. Additionally, the gut microbiota can regulate the production, function, and maturation of neutrophils, and neutrophils also influence the composition and function of the microbiota. These two factors interact and jointly affect the development of IBD [50].

CD4 + T cells display distinct characteristics in CD and UC. In CD patients, there is a significant expansion of CD4 + tissue-resident memory T (Trm) cells in the gut. Notably, CD4 + Trm subsets expressing CD161 and CCR5 in CD patients exhibit stronger cytotoxicity and are associated with disease activity. In contrast, CD45RA + CCR7 + naive CD4 + T cells and CXCR5 + T follicular helper (Tfh) cells are abundant in the intestines of UC patients. Moreover, CD4 + Trm (CDtrm) cells possess innate-like cell properties. These cells can rapidly secrete inflammatory cytokines such as IFN-γ upon cytokine stimulation without the need for T-cell receptor (TCR) activation. This ability can cause damage to intestinal epithelial cells. In UC, the increase in Tfh cells may be linked to the increase in pathological IgG + plasma cells [51].

Currently, in clinical practice, achieving an accurate diagnosis of IBD remains a challenge. The diagnosis relies mainly on medical history, clinical symptoms, laboratory tests, imaging examinations, endoscopy, and histological examinations. Colonoscopy is widely regarded as the “gold standard” for diagnosing IBD. However, colonoscopy is time-consuming and highly dependent on the diagnostic experience of relevant physicians. Additionally, the intestinal wall of IBD patients in severe stages is extremely fragile, and improper procedures during colonoscopy can lead to complications such as bleeding and perforation. Therefore, our objective is to identify more reliable and sensitive biomarkers that can directly reflect the different stages of IBD and its underlying disease mechanisms. To this end, we carried out rigorous screening in databases such as the GEO, IBDDMB, and the UKB. We imposed strict restrictions on the number of patients included in the study. Only patients from cohorts with a population size greater than 100 were considered, and the scope of inclusion covered all stages of IBD onset and the entire intestinal tissue. Unfortunately, despite obtaining a large amount of data from public databases for analysis and striving to include as much complete data as possible for real-world validation, our sample size is too small to rule out the possibility of data randomness. However, we have conducted multiple experiments to minimize errors as much as possible. Notably, we conducted a comprehensive analysis of existing IBD data by multiple methods and genetic-based research on specific immune cells exhibiting differences in the immune response, which is unprecedented. This method provides a new research perspective for the study of IBD.

Currently, ANNs, machine learning, interpretable analysis, and multichip joint analysis are gaining popularity in medical research. Given the complex genetic basis of IBD and the limitations of existing diagnostic tools, the diagnosis of IBD often poses significant challenges. By analyzing extensive genomic data, ANNs and machine learning hold great promise for increasing diagnostic efficiency and accuracy. They can also identify complex multiallele patterns that may be associated with specific diseases. Through interpretable analysis, detailed insights can be provided into the contribution of DEGs within diagnostic models, thereby offering explanations for machine learning models [52]. The application of artificial neural networks and computer technologies such as machine learning and deep learning to assist clinical doctors in disease diagnosis and treatment has become increasingly prevalent. Continuously evolving artificial intelligence enables more precise and scientific diagnosis and treatment, ultimately benefiting patients in need of medical care.

## Conclusion

In this study, we developed a diagnostic model for predicting IBD with a comprehensive ANN, machine learning, interpretable analysis, and multichip joint analysis methods. ANN, LASSO, SVM, and random forest algorithms were used for genetic feature selection. We identified four key biomarkers (LOC389023, DUOX2, LCN2, and DEFA6) and used ten machine learning methods and SHAP models to assist in IBD diagnosis, clarifying the genetic characteristics, molecular pathways, and differentially abundant immune cells of IBD. These genes were demonstrated to be related to immune system function. In addition, we conducted multiple validations with the dataset. Our findings indicate that machine learning algorithms can facilitate accurate diagnostic decisions for IBD, enabling clinicians to explore new treatment pathways and diagnostic methods.

## Electronic supplementary material

Below is the link to the electronic supplementary material.

Supplementary Material 1

Supplementary Material 2

Supplementary Material 3

Supplementary Material 4

Supplementary Material 5

Supplementary Material 6

Supplementary Material 7

Supplementary Material 8

## Data Availability

The GSE87466, GSE179285, and GSE87473 microarray datasets used in this study were downloaded from the Gene Expression Omnibus database.
