# Supplementary figures and images for: Construction of a feature gene and machine prediction model for inflammatory bowel disease based on multichip joint analysis
Source: J Transl Med. 2025 Aug 19;23:937. doi: 10.1186/s12967-025-06838-z (PMC12366088; doi:10.1186/s12967-025-06838-z)

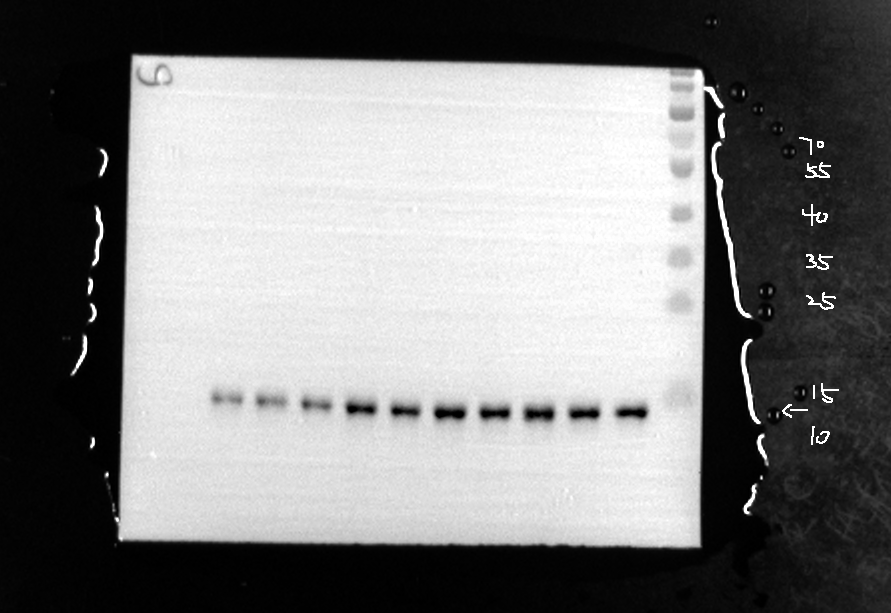

Supplement: Supplementary file 1 — Supplementary Material 1 [file 12967_2025_6838_MOESM1_ESM.tif]

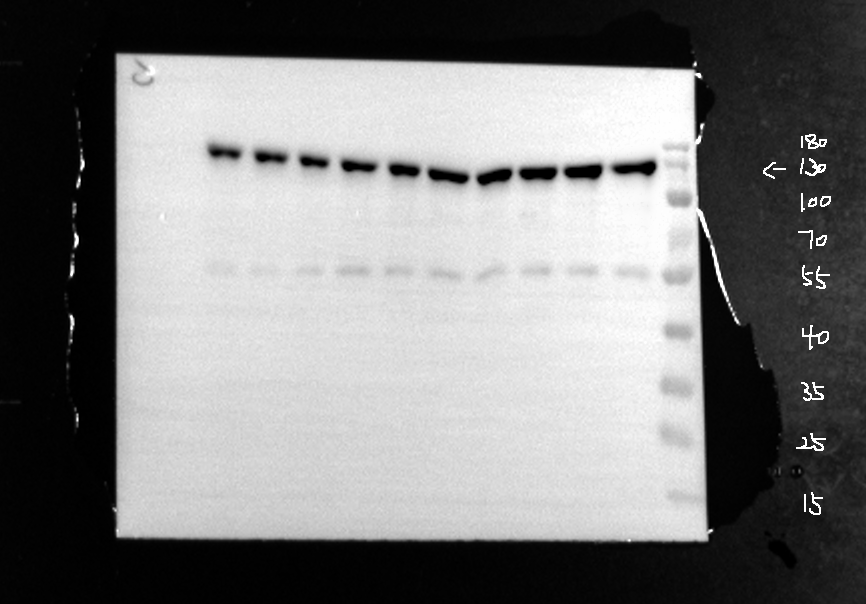

Supplement: Supplementary file 2 — Supplementary Material 2 [file 12967_2025_6838_MOESM2_ESM.tif]

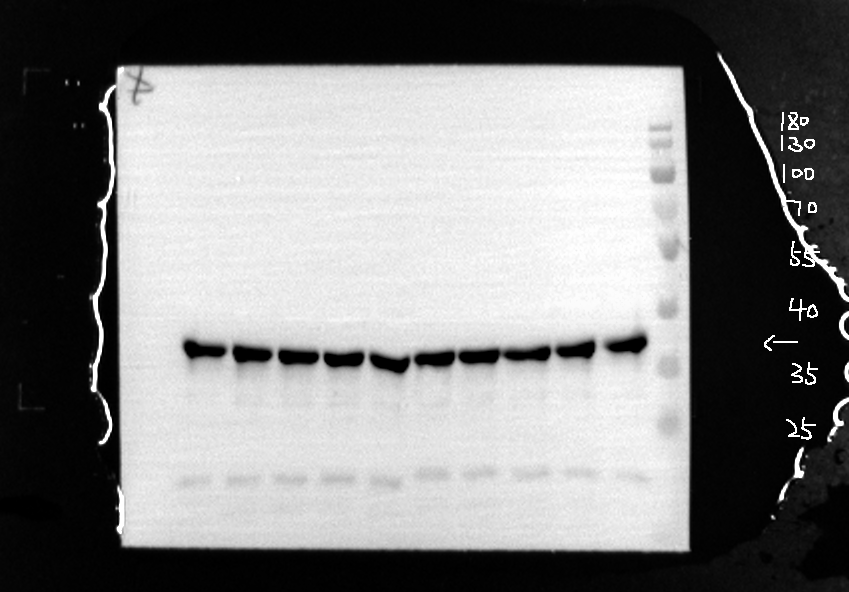

Supplement: Supplementary file 3 — Supplementary Material 3 [file 12967_2025_6838_MOESM3_ESM.tif]

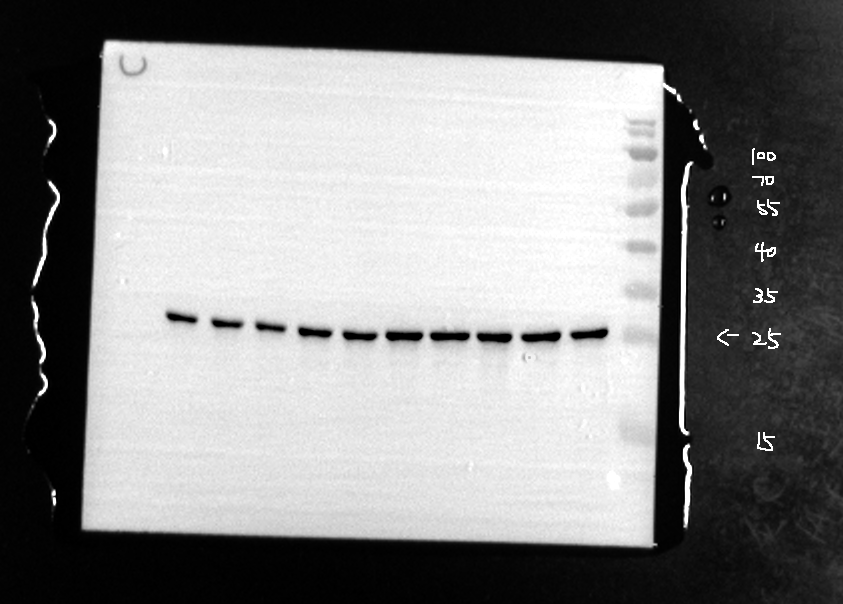

Supplement: Supplementary file 4 — Supplementary Material 4 [file 12967_2025_6838_MOESM4_ESM.tif]

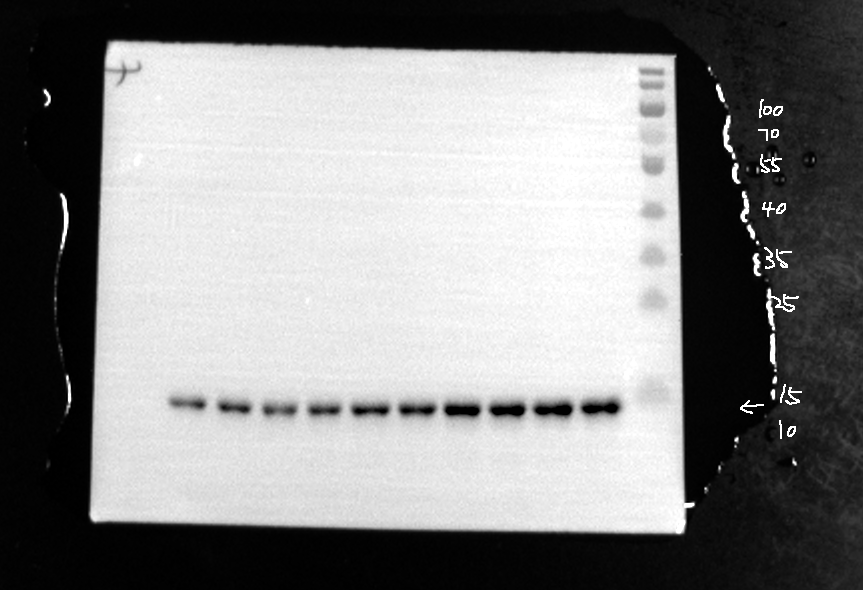

Supplement: Supplementary file 5 — Supplementary Material 5 [file 12967_2025_6838_MOESM5_ESM.tif]

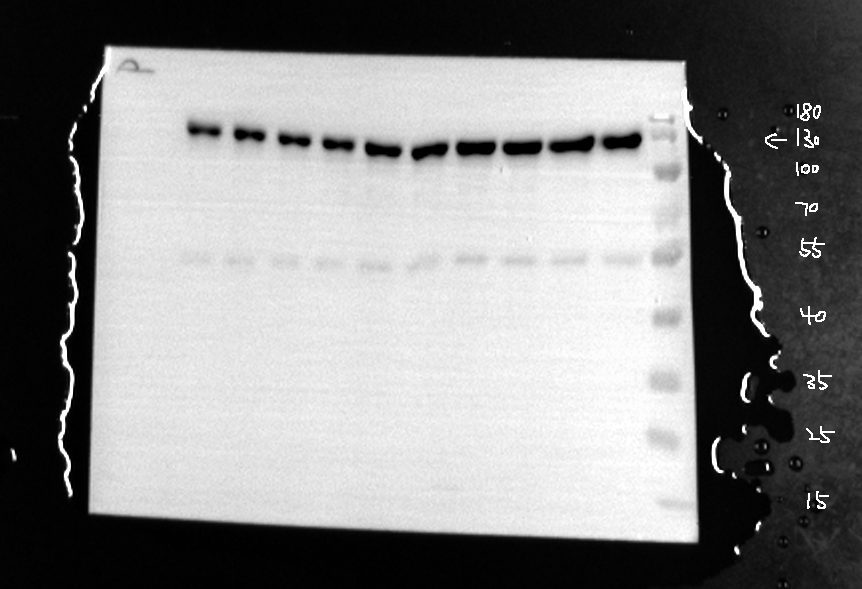

Supplement: Supplementary file 6 — Supplementary Material 6 [file 12967_2025_6838_MOESM6_ESM.tif]

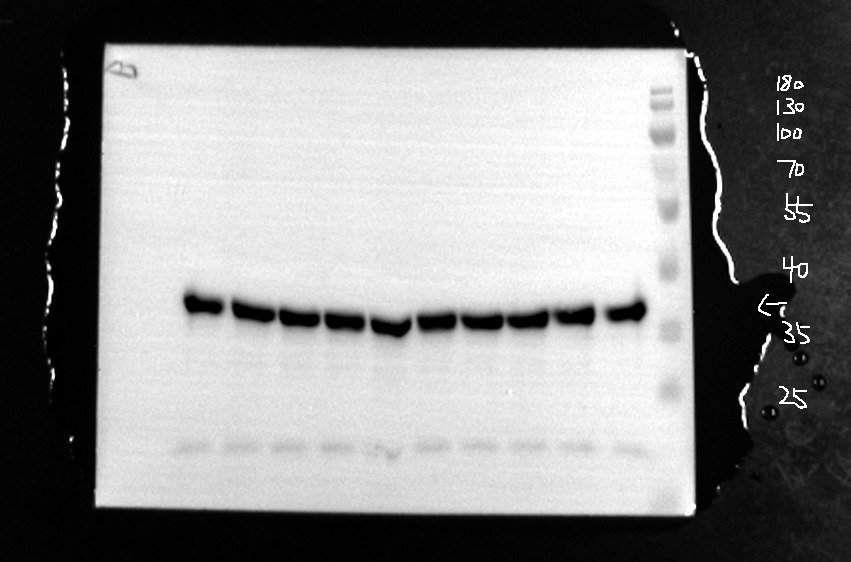

Supplement: Supplementary file 7 — Supplementary Material 7 [file 12967_2025_6838_MOESM7_ESM.tif]

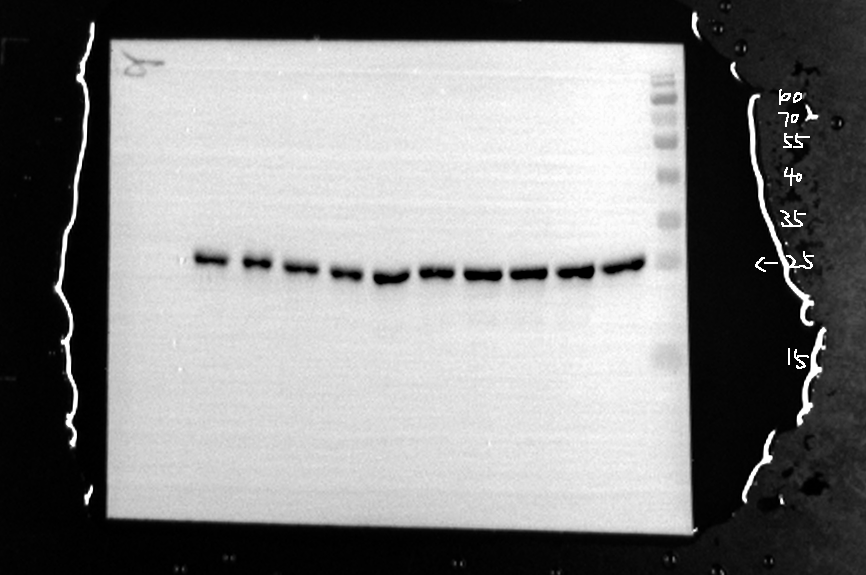

Supplement: Supplementary file 8 — Supplementary Material 8 [file 12967_2025_6838_MOESM8_ESM.tif]
